# Peripheral T cell receptor diversity is associated with clinical outcomes following ipilimumab treatment in metastatic melanoma

**DOI:** 10.1186/s40425-015-0070-4

**Published:** 2015-06-16

**Authors:** Michael A. Postow, Manuarii Manuel, Phillip Wong, Jianda Yuan, Zhiwan Dong, Cailian Liu, Solène Perez, Isabelle Tanneau, Marlène Noel, Anaïs Courtier, Nicolas Pasqual, Jedd D. Wolchok

**Affiliations:** Memorial Sloan Kettering Cancer Center, 1275 York Avenue, New York, NY 10065 USA; Weill Cornell Medical College, 525 E 68th Street, New York, 10065 USA; ImmunID, Grenoble, France; Immune Monitoring Core Facility, Ludwig Center for Cancer Immunotherapy, Memorial Sloan Kettering Cancer Center, New York City, NY 10065 USA; Assistant Attending Physician, Melanoma and Immunotherapeutics Oncology Service, Memorial Sloan Kettering Cancer Center, 1300 East 66th Street, New York, NY 10065 USA

## Abstract

**Background:**

Ipilimumab improves overall survival in a subset of patients with metastatic melanoma. Peripheral blood T cell receptor (TCR) repertoire diversity has been associated with favorable outcomes in patients with cancer, but its relevance as a biomarker for ipilimumab outcomes remains unknown.

**Findings:**

In this pilot study, we analyzed the pre-treatment peripheral blood TCR repertoire in 12 patients with metastatic melanoma who received ipilimumab at 3 mg/kg (clinical benefit, n = 4; no clinical benefit, n = 8). TCR diversity was evaluated using a polymerase chain reaction assay which measures TCR combinatorial diversity between V and J genes from genomic DNA. TCR repertoire diversity was studied through richness (observed V-J rearrangements) and evenness (similarity between the frequencies of specific V-J rearrangements). The Wilcoxon rank sum test was used to compare patients with clinical benefit and those without. Association with benefit in a dichotomized analysis was assessed through a Fisher’s exact test. Overall survival was studied through log-rank analysis.

There was a significant difference in richness (p = 0.033) and evenness (p = 0.028) between patients with and without clinical benefit. Dichotomized analysis showed that none of the patients with low richness (n = 0/5, p = 0.081) nor low evenness (n = 0/7, p = 0.01) achieved clinical benefit. There were no significant differences in overall survival.

**Conclusions:**

In this small group of patients, baseline TCR diversity in the peripheral blood was associated with clinical outcomes. Further investigation is ongoing in larger cohorts of patients to explore these preliminary findings and determine whether TCR diversity can be used as a predictive biomarker in cancer immunotherapy.

**Electronic supplementary material:**

The online version of this article (doi:10.1186/s40425-015-0070-4) contains supplementary material, which is available to authorized users.

## Background

Antibodies that block immunologic checkpoints can result in long-lasting benefit for patients with many different malignancies. Cytotoxic T lymphocyte antigen 4 (CTLA-4) was the first immunologic checkpoint to be clinically targeted, and the anti-CTLA-4 antibody, ipilimumab, has been shown to improve overall survival in a subset of patients [1, 2]. Identifying which patients are most likely to benefit from ipilimumab remains an active area of investigation.

Since ipilimumab is believed to exert its antitumor effects through T cells, a number of studies have investigated T lymphocyte populations and correlated immunologic changes with patient outcomes. Increases in the absolute lymphocyte count and markers of activation such as inducible co-stimulator (ICOS) on CD4+ T cells have been shown to be pharmacodynamic biomarkers of ipilimumab that correlate with overall survival [3-7]. Other investigations have focused on the peripheral blood T-cell receptor (TCR) repertoire and have shown that CTLA-4 blockade diversifies the TCR repertoire [8]. In another study, maintenance of high-frequency TCR clonotypes during CTLA-4 blockade was associated with improved overall survival [9].

To better determine whether the pre-treatment TCR repertoire diversity was associated with clinical outcomes following ipilimumab, we conducted a pilot study of 12 patients with metastatic melanoma treated with ipilimumab. Since a diverse TCR repertoire may increase the likelihood that a relevant antitumor T cell population is present and has been associated with favorable outcomes in patients with other malignancies such as breast cancer [10], we hypothesized that the combinatorial diversity of the TCR repertoire would be relevant to clinical outcomes following ipilimumab.

## Findings

### Patients and treatment

Twelve patients with metastatic melanoma who were treated with ipilimumab were selected for inclusion in this analysis based upon sample availability and annotated clinical data. Clinical benefit was determined by evidence of tumor burden reduction or prolonged stable disease lasting at least 9 months following initiation of ipilimumab. Eight patients had no apparent clinical benefit from ipilimumab, and four patients had clinical benefit. All patients received ipilimumab at 3 mg/kg as per standard of care outside of a clinical trial. All patients provided informed consent to an institutional review board (IRB) approved correlative blood drawing research protocol prior to the collection of peripheral blood (Memorial Sloan Kettering Cancer Center IRB # 00-144).

### Assessment of T cell receptor repertoire combinatorial diversity

Peripheral blood was collected and stored as previously described [11]. T cell receptor (TCR) diversity was evaluated by authors blinded to clinical outcome using the ImmunTraCkeR® test (ImmunID), a multiplex polymerase chain reaction (PCR) assay which measures combinatorial diversity of the TCR beta chain. Genomic DNA was extracted from blood clots using the QIAamp DNA Blood Mini Kit (Qiagen) and concentrated using Amicon Ultra-0.5 mL Centrifugal Filters (EMD Millipore). Multiplex PCR was performed using an upstream primer specific for all functional members of a given V family and a downstream primer specific for a J segment. This assay allows the simultaneous detection of all TRBV–TRBJ rearrangements covering 100 % of the possible combinatorial rearrangements (based on the international ImMunoGeneTics information system®, IMGT®, http://www.imgt.org). PCR products were separated by microfluidic migration (Labchip GX, Perkin Elmer) using a lab-on-a-chip (HT DNA 12 K LabChip Kit, Perkin Elmer). All V–J1, J2, J3, J4, Jn products were separated as a function of their size with a maximum amplicon expected size of ~5 kb.

The Constel’ID® software (ImmunID) was used to analyze the data produced during the microfluidic migration and to generate a three-dimensional immune map. This map is composed of peaks of variable height corresponding to the rearrangements actually observed in one sample with the height of each peak depending upon the frequency of a particular TCR rearrangement. (Example patient shown in Additional file 1: Figure S1).

Two separate metrics were used to further characterize the diversity of the TCR repertoire—richness and evenness. Richness reflects how many different V-J rearrangements are present and is defined as the ratio (expressed as a percentage) between the number of observed rearrangements in a sample and the number of possible theoretical rearrangements between V families and J genes. Evenness reflects how similar the frequencies of V-J rearrangements are to each other. An evenness value for each patient in this study was calculated as the ratio of how many rearrangements amongst the most frequent were necessary to account for 50 % of the global map intensity (cumulative sum of each rearrangement’s frequency) divided by the total number of rearrangements present. High evenness corresponds to V-J rearrangements evenly represented in terms of frequency. Low evenness corresponds to a TCR repertoire dominated by specific V-J rearrangements that may indicate increased clonality.

### Hierarchical clustering

Similarity among patients’ baseline samples was assessed based on correlation between TCR repertoires, using each single V-J rearrangement contribution. Unsupervised hierarchical clustering with multiscale bootstrap resampling (n = 10,000) was then performed to group the most similar samples in a dendrogram. The measure of distance used for clustering was based on correlation between immune profiles, using each single rearrangement contribution. Ward method was employed as the method of agglomeration. Approximately unbiased (AU) probability values were calculated based on multiscale bootstrap resampling.

### Association of T cell receptor repertoire diversity (richness and evenness) with clinical outcome

Both richness and evenness were compared as a continuous variable between patients with and without clinical benefit using a Wilcoxon rank sum test. A receiver operating characteristic (ROC) curve was used to determine a possible threshold for dichotomized analysis (i.e. low vs. high diversity richness/evenness). Association with benefit in the low vs. high groups was assessed through a Fisher’s exact test. Overall survival was studied through log-rank analysis. Statistical analyses were performed using the R software.

### Patients and treatment

A total of twelve patients with stage IV melanoma were included in this analysis as described in Table 1. All had a Karnofsky Performance Status of 90-100 %. Only one patient with clinical benefit had a baseline LDH available as indicated. There were no obviously apparent demographic differences between patients with and without clinical benefit in this small, non-randomized dataset. The median follow-up time for overall survival was 785 days.

**Table 1:** Patient characteristics

	Clinical benefit (n = 4)	No clinical benefit (n = 8)
Median age (range)	57 (38-78)	67 (52-77)
Sex	1 female	3 female
	3 male	5 male
M-stage	0 M1A	2 M1A
	2 M1B	0 M1B
	2 M1C	6 M1C
Median baseline LDH	156	211 (183-438)
Karnofsky Performance	90 (90-100)	90 (90-100)

### Association of T cell receptor repertoire diversity with clinical outcomes

**Fig. 1:**
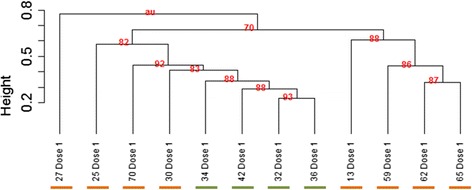
Hierarchical clustering of 12 patients’ TCR repertoires using each single V-J rearrangement contribution. Red values indicate approximately unbiased (AU) probability values

Unsupervised hierarchical clustering that compared VJ usage among all of the patients revealed that the four patients with clinical benefit (green) had similar VJ usage (Fig. 1). Four of the eight patients who did not achieve clinical benefit (orange, right side of Fig. 1) also appeared to cluster together. There were no obviously apparent clinical differences between the four patients without benefit who clustered separately (orange, left side of Fig. 1) from the other four patients without benefit (orange, right side of Fig. 1).

**Fig. 2:**
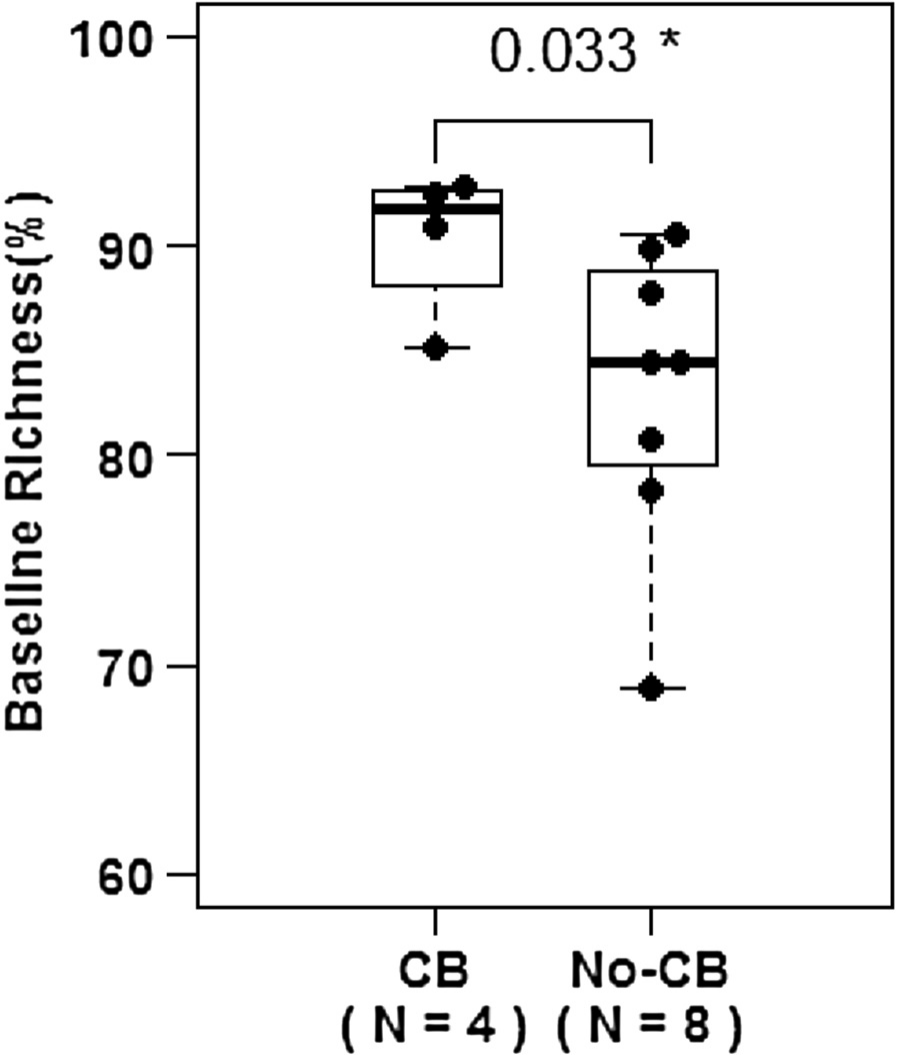
Patients who had clinical benefit had a higher degree of TCR repertoire richness at baseline (p = 0.033)

Based upon these findings from hierarchical clustering analysis that revealed similarity in VJ usage among the patients with clinical benefit, we next sought to evaluate whether TCR diversity (richness and evenness) was associated with clinical benefit. Patients who experienced clinical benefit from ipilimumab had a higher degree of richness as a continuous variable than patients who did not achieve clinical benefit (Fig. 2, p = 0.033). To further explore this finding of TCR repertoire richness, a ROC curve was created, and a threshold of 85 % was selected to dichotomize patients into “low vs. high” richness groups (Additional file 2: Figure S2). None of the patients with “low” richness (n = 5) experienced clinical benefit, but four out of the seven patients with “high” richness experienced clinical benefit (p = 0.081). There was no significant difference in overall survival between patients with “low” vs. “high” richness (p = 0.218).

**Fig. 3:**
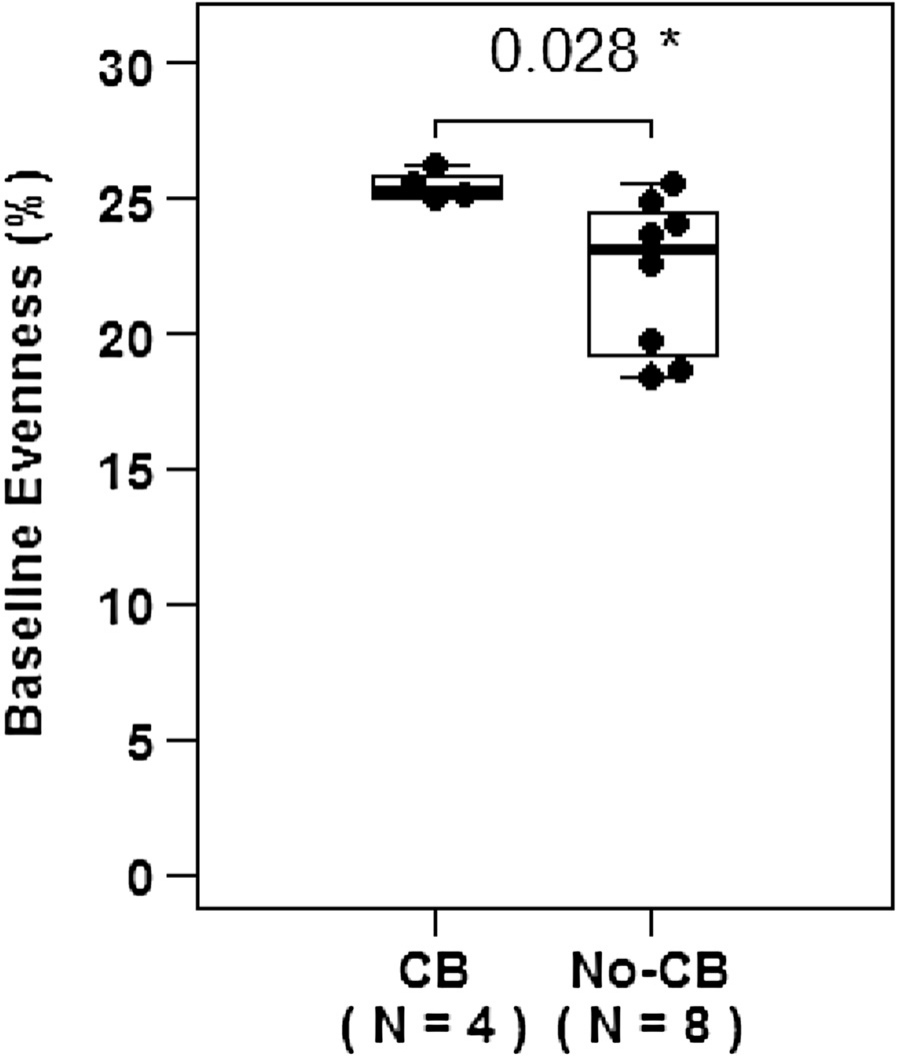
Patients who had clinical benefit had a higher degree of TCR repertoire evenness at baseline (p = 0.028)

Patients who experienced clinical benefit from ipilimumab had a higher degree of evenness as a continuous variable compared to patients who did not achieve clinical benefit (Fig. 3, p = 0.028). A ROC curve was created for TCR repertoire evenness, and a threshold of 25 % was selected to dichotomize patients into “low vs. high” evenness groups (Additional file 3: Figure S3). None of the patients with “low” evenness (n = 7, example patient Additional file 4: Figure S4A) experienced clinical benefit, whereas four out of the five patients with “high” evenness (example patient Additional file 4: Figure S4B) experienced clinical benefit (p = 0.01). There was no significant difference in overall survival between patients with “low” vs. “high” evenness (p = 0.26).

## Discussion

In a small group of patients with advanced melanoma, TCR diversity in the peripheral blood, as assessed by this specific technology, prior to treatment with ipilimumab was shown to correlate with clinical outcomes. Patients who had clinical benefit had a higher degree of richness and evenness in their baseline TCR repertoires than patients who did not have clinical benefit. Our results suggest that an immunologically diverse TCR repertoire prior to treatment may be important to optimizing outcomes with ipilimumab.

Prior studies have evaluated the diversity within the TCR repertoire during treatment with CTLA-4 blockade using next-generation sequencing platforms that sequence the TCR complementarity determining region 3 (CDR3) [8, 9, 12]. Several of these publications examined “on treatment” changes in TCR diversity during CTLA-4 blockade which we could not investigate due to limited sample availability [9, 12]. In contrast to our findings, one of these prior studies found no association between baseline TCR repertoire diversity and response to CTLA-4 blockade with tremelimumab [8]. It is unclear why our results differ, but differences in CTLA-4 blocking antibodies (tremelimumab in [8] and ipilimumab in this study) and differences in technology are possible explanations. Since many different technologies are currently being investigated to assess the TCR repertoire, until additional validation is obtained for each, it remains to be determined which methodology will be the most robust for future investigations.

Our study is inherently limited by its small size and retrospective nature and is therefore considered hypothesis generating. Since these patients were not treated on clinical trials, rigorous tumor measurements such as Response Evaluation Criteria in Solid Tumors (RECIST) were not applied. Prior to the examination of TCR repertoire diversity, clinical benefit was determined independently by study investigators who retrospectively categorized patient outcomes based upon evidence of tumor shrinkage or prolonged stable disease. The peripheral blood TCR diversity data were then generated by separate individuals who were blinded to the patients’ clinical outcomes.

Our findings showed that TCR diversity was associated with clinical benefit but not overall survival. One possible explanation for this could be the long survival times of some of the patients in the group without clinical benefit to ipilimumab. For example, one patient who was alive 785 days after initiating ipilimumab progressed in the brain with oligometastatic disease that was successfully treated with stereotactic radiosurgery. Another patient with 991 days of survival after initiating ipilimumab progressed on ipilimumab but achieved subsequent benefit with an antibody that blocks the programmed death ligand 1. It is also possible that the overall sample size was too small to detect a statistically significant difference between TCR diversity and overall survival.

As this was a small cohort, the thresholds we established for “low” vs. “high” richness and evenness groups are considered exploratory. As additional patients are studied, the sensitivity and specificity of various thresholds will become more confidently established. Despite the small sample size, the diversity of the TCR repertoire (richness per continuous analysis and evenness per both continuous and dichotomized analysis) was significantly associated with ipilimumab benefit.

A baseline TCR repertoire containing many different V-J rearrangements (richness) represented at evenly distributed frequencies (evenness) with no one particular skewing of a specific set of clones may be favorable because it could indicate an increased likelihood that a particular immunologically “relevant” antitumor T cell population is present that, once released from suppression by CTLA-4 blockade, can expand and contribute to a tumor response. A more diverse TCR repertoire may also be reflective of better “immunologic health” in general. A diverse TCR repertoire has previously been shown to be associated with decreased infection risk after allogeneic stem cell transplantation [13], and as we have shown, now appears potentially relevant to outcomes following ipilimumab. A large multicenter study (Predict-ID Melanoma, France) for confirmation of these preliminary findings and for evaluation of changes in TCR repertoire during treatment is underway.

## Additional files

Additional file 1: Figure S1. Example of a patient’s immune profile. The X-Y axis reflects the presence of a particular V-J recombination event. Richness is dependent upon the number of specific V-J rearrangements present. The Z-axis reflects the frequency of a particular recombinant which is used to calculate evenness. Additional file 2: Figure S2. A threshold of 85 % was selected to dichotomize patients into low and high richness groups based upon sensitivity and specificity calculations. Additional file 3: Figure S3. A threshold of 25 % was selected to dichotomize patients into low and high evenness groups based upon sensitivity and specificity calculations. Additional file 4: Figure S4. An example patient with low evenness <25 % (A) and high evenness ≥25 % (B).

## Abbreviations

TCR: T cell receptor
CTLA-4: Cytotoxic T lymphocyte antigen-4
PCR: Polymerase chain reaction
