# Supplementary figures and images for: Peripheral T cell receptor diversity is associated with clinical outcomes following ipilimumab treatment in metastatic melanoma
Source: J Immunother Cancer. 2015 Jun 16;3:23. doi: 10.1186/s40425-015-0070-4 (PMC4469400; doi:10.1186/s40425-015-0070-4)

## Slide 1
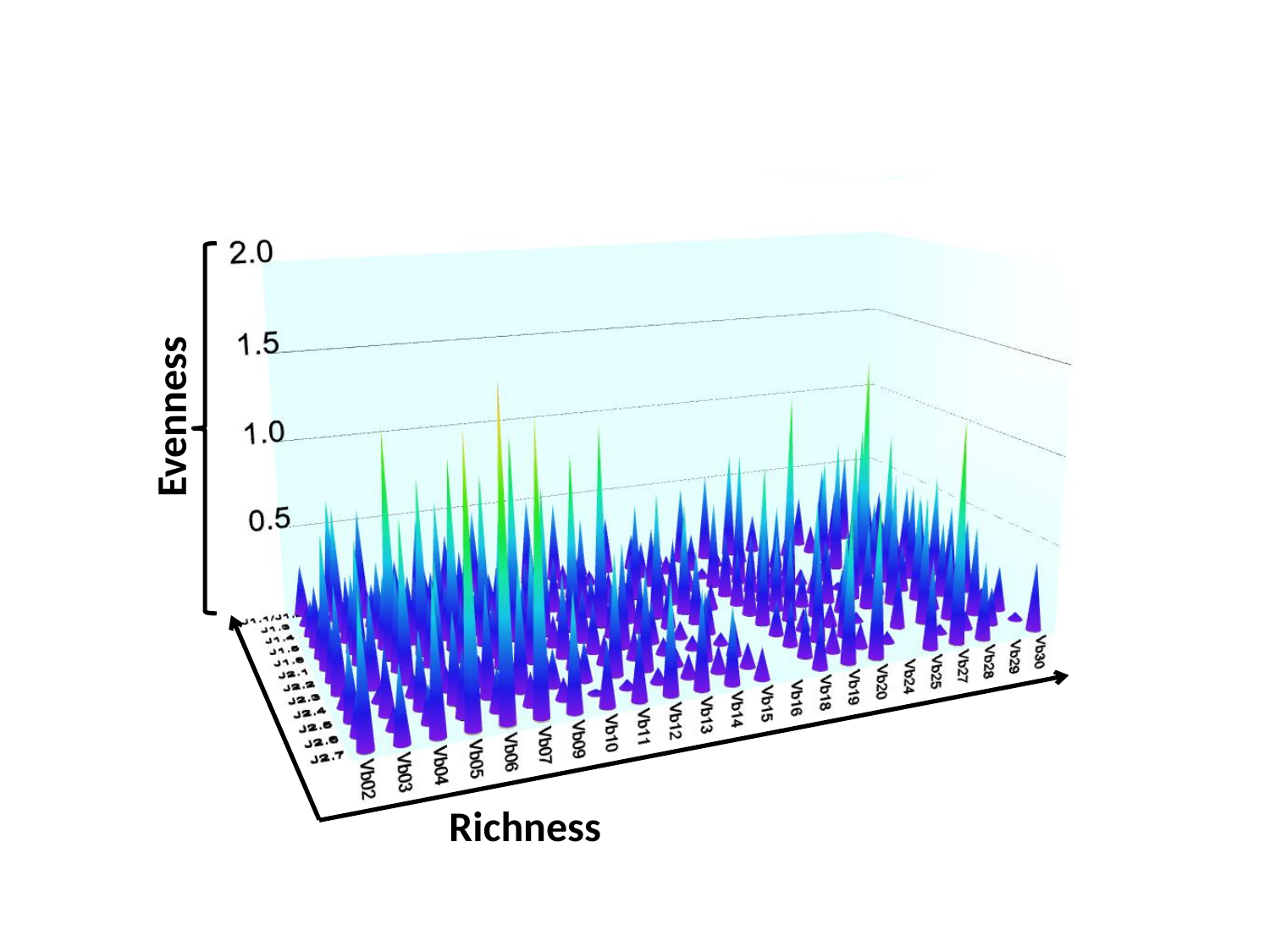

Evenness
Richness

Supplement: Additional file 1: Figure S1. — Example of a patient’s immune profile. The X-Y axis reflects the presence of a particular V-J recombination event. Richness is dependent upon the number of specific V-J rearrangements present. The Z-axis reflects the frequency of a particular recombinant which is used to calculate evenness. [file 40425_2015_70_MOESM1_ESM.pptx]

## Slide 1
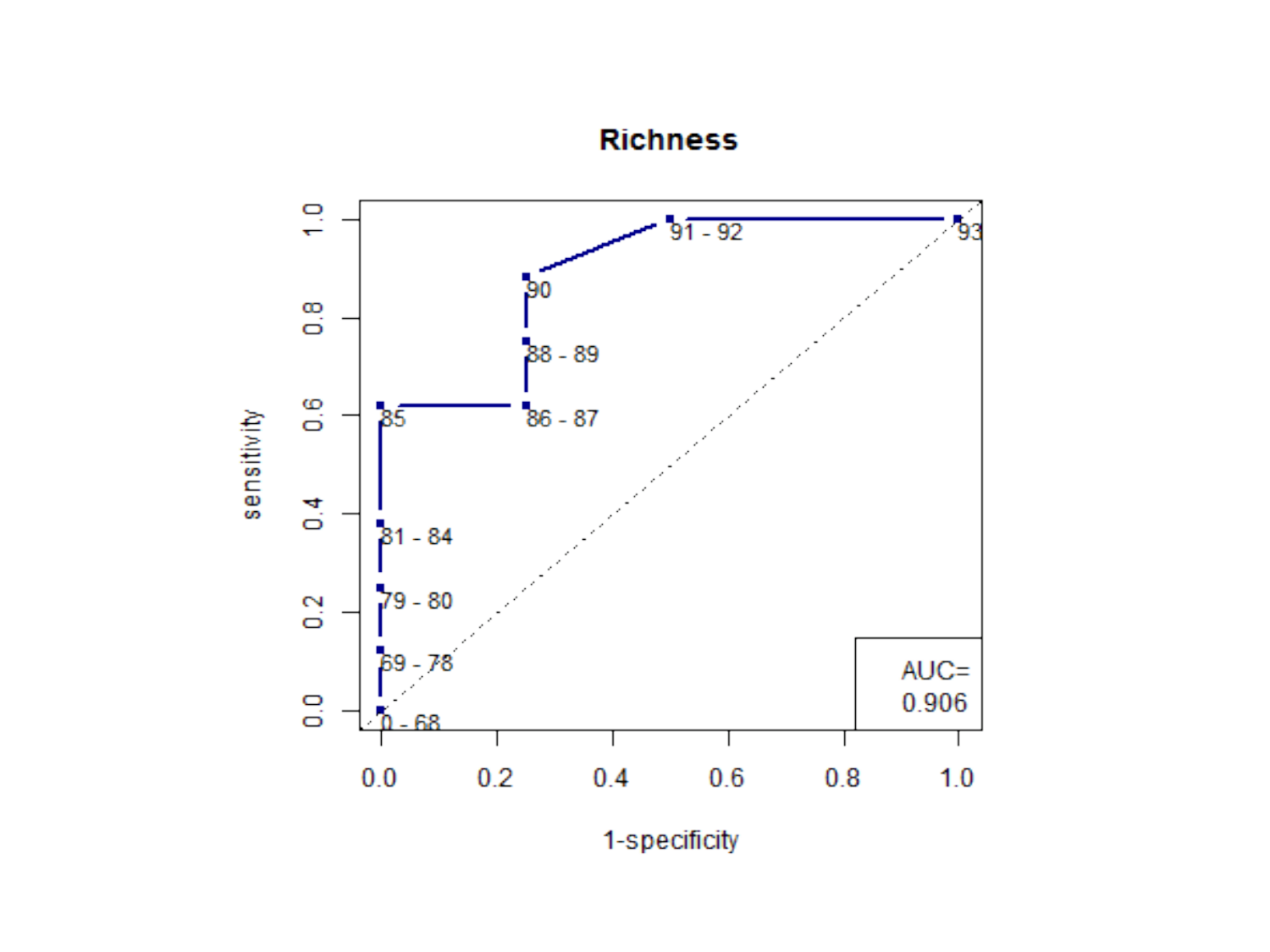

Supplement: Additional file 2: Figure S2. — A threshold of 85 % was selected to dichotomize patients into low and high richness groups based upon sensitivity and specificity calculations. [file 40425_2015_70_MOESM2_ESM.pptx]

## Slide 1
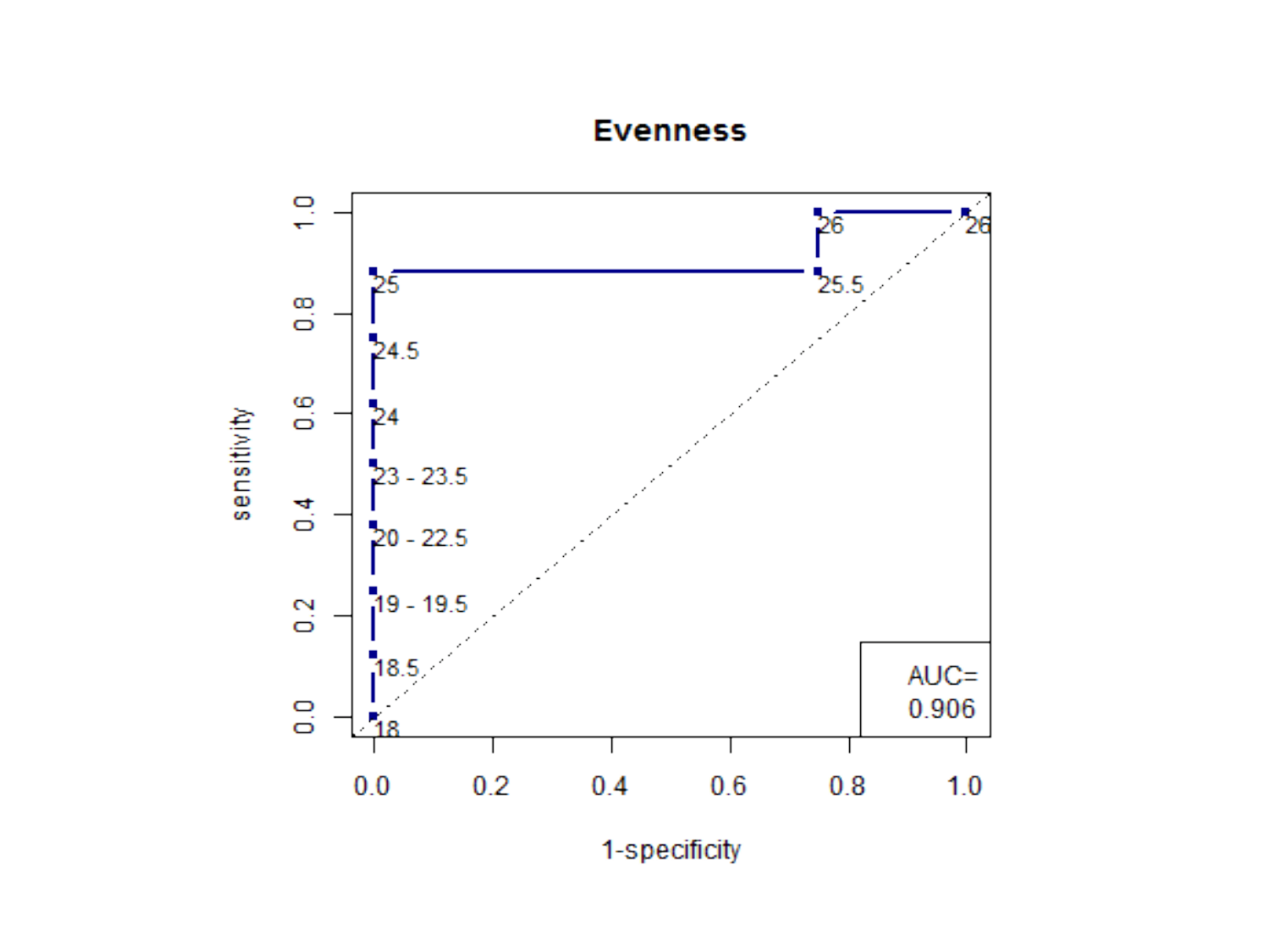

Supplement: Additional file 3: Figure S3. — A threshold of 25 % was selected to dichotomize patients into low and high evenness groups based upon sensitivity and specificity calculations. [file 40425_2015_70_MOESM3_ESM.pptx]

## Slide 1
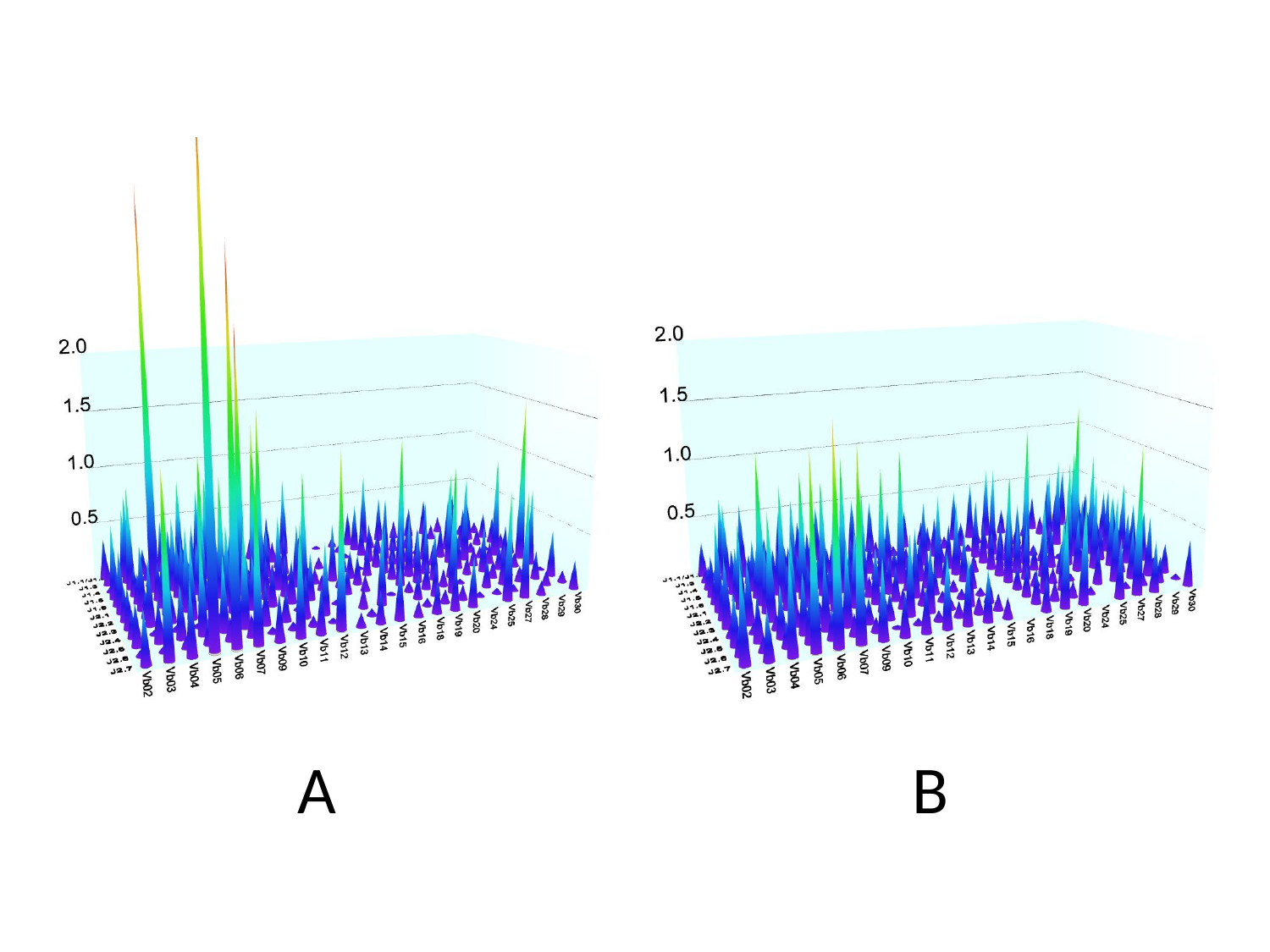

A
B

Supplement: Additional file 4: Figure S4. — An example patient with low evenness <25 % (A) and high evenness ≥25 % (B). [file 40425_2015_70_MOESM4_ESM.pptx]
